# Supplementary material for: Regulation of mRNA translation during mitosis
Source: eLife. 2015 Aug 25;4:e07957. doi: 10.7554/eLife.07957 (PMC4548207; doi:10.7554/eLife.07957)
Supplement: Supplementary file 1. — Quality control of the deep sequencing data set. (A) The number of reads for the two biological replicates of the G2 FP sample was plotted on a log2 scale. Only genes with an average of at least 100 reads per replicate where selected for subsequent analysis. The box marked off by the two black lines indicates the genes that were excluded from the analysis, as they had less than 200 reads total. (B) After filtering the data (see ‘Materials and methods’) the ratio of the RPKM values was determined for all genes of each set of replicates. The standard deviation was determined for each set of replicates. The data sets of the two replicates were also fit and the R2 for the fit was determined. DOI: http://dx.doi.org/10.7554/eLife.07957.014 [file elife07957s001.pdf]

Supplementary file 1. Quality control of the deep sequencing data set.

A

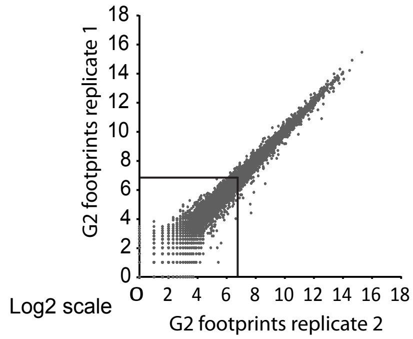

B

	Rsquared	SD
**G2 mRNA**	0.82	0.333289
**G2 FP**	0.98	0.273472
**M mRNA**	0.95	0.196312
**M FP**	0.96	0.259008
**G1 mRNA**	0.97	0.175126
**G1 FP**	0.96	0.267667
